# Uptake, Retention, and Excretion of Infectious Prions by Experimentally Exposed Earthworms

**DOI:** 10.3201/eid2712.204236

**Published:** 2021-12

**Authors:** Sandra Pritzkow, Rodrigo Morales, Manuel Camacho, Claudio Soto

**Affiliations:** University of Texas Health Science Center at Houston, Houston, Texas, USA (S. Pritzkow, R. Morales, M. Camacho, C. Soto);; CIBQA, Universidad Bernardo O’Higgins, Santiago, Chile (R. Morales);; Case Western Reserve University, Cleveland, Ohio, USA (M. Camacho)

**Keywords:** prions and related diseases, chronic wasting disease, environmental transmission, PMCA, earthworms

## Abstract

Prions are proteinaceous infectious agents that can be transmitted through various components of the environment, including soil particles. We found that earthworms exposed to prion-contaminated soil can bind, retain, and excrete prions, which remain highly infectious. Our results suggest that earthworms potentially contribute to prion disease spread in the environment.

Prions are unique infectious agents composed exclusively of a misfolded form of the prion protein (PrP^Sc^) (*1*). Among prion diseases, chronic wasting disease, affecting cervids, and scrapie, affecting sheep, are highly contagious. Studies conducted in natural and experimental conditions suggest that these diseases likely are transmitted via environmental contamination and that soil is a primary vector (*2*–*4*). We examined whether earthworms contribute to environmental spread of infectious prions.

## The Study

To investigate whether earthworms can act as carriers of infectious prions, we exposed groups of worms (*Eisenia fetida*) to soil previously mixed with brain homogenate (BH) from clinically diseased 263K Syrian golden hamsters (*Mesocricetus auratus*) (Harlan Envigo, https://www.envigo.com). For experiments, we homogenously mixed 375 g of Elliot soil (kindly provided by Joel Pedersen, Johns Hopkins University) with 25 mL of 10% wt/vol 263K brain homogenate. We assessed whether prions bind to worms or worm-associated soil by using protein misfolding cyclic amplification (PMCA) technology (*5*,*6*), which can detect prions down to the level of a single particle (*7*). Because PMCA efficiency can be severely affected by components in the inoculum (*6*), we first analyzed the effect of worm homogenate (WH) with or without soil on the efficiency of in vitro prion replication by PMCA (Appendix Figure 1). Our results indicated that whole WH does interfere with the reaction, but we could still obtain maximum amplification after 3 rounds of PMCA (Appendix Figure 1). 

**Figure 1:**
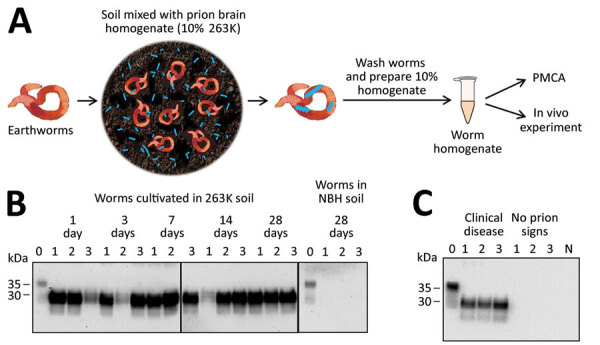
Detection of prion protein (PrP^Sc^) attached to earthworms by PMCA and infectivity bioassay. A) Process for exposing earthworms to infected soil. Earthworms were placed in soil mixed with 10% wt/vol infected 263K hamster brain homogenate for 1, 3, 7, 14, or 28 days; worms were washed thoroughly, then prepared into a 10% homogenate for analysis. B) Results of PMCA on earthworms exposed to contaminated soil. As a control, earthworms also were exposed to soil mixed with NBH for 28 days and analyzed with the same methods. For each measurement, 3 worms were analyzed per time point in 3 different gels but blotted in the same membrane. Lane 0 is NBH used as a positive control for electrophoretic migration of the normal prion protein (PrP^C^); lanes 1–3 indicate 3 different worms. Vertical lines between images depict membrane splicing. Numbers on the left indicate molecular weight markers. C) Biochemical analysis of brains of hamsters infected with worm homogenate. Groups of hamsters were injected with homogenates from 3 different worms exposed to prion contaminated soil; many of the animals developed prion disease (Appendix Figure 2). Brains were collected and homogenized and samples were digested with proteinase K (Sigma Aldrich, https://www.sigmaaldrich.com) at 50 µg/mL for 1 h at 37°C, except NBH (lane labeled N) used as a migration control. Numbers on the left indicate molecular weight markers. Results confirmed the presence of PrP^Sc^ accumulation in the brain of animals showing clinical signs of prion disease. NBH, normal hamster brain homogenate; PMCA, protein misfolding cyclic amplification.

After verifying PMCA efficiency, we tested worms exposed to contaminated soil for different lengths of time. We collected worms from contaminated soil after 1, 3, 7, 14, and 28 days of exposure (Figure 1, panel A). PMCA results showed that worms exposed to prions take up PrP^Sc^ and efficiently sustain prion replication at all exposure times tested (Figure 1, panel B). We observed no PrP^Sc^ uptake in any worms exposed to control soil.

To study whether contaminated worms can transmit disease, we intraperitoneally injected hamsters with WH obtained from worms exposed to prion-soil mix for 28 days. To assess reproducibility, we used 3 different worms for this assay. Our results showed that worms exposed to prion-contaminated soil can transmit prion disease, albeit with variable efficiencies (Appendix Figure 2). Of the 3 worm extracts, 2 caused an attack rate of 4/5 and mean incubation periods of 237 (SE +39) and 255 (SE +25) days. A third WH transmitted disease to only 1/5 injected hamsters, which showed an incubation period of 272 days (Appendix Figure 2). For positive controls, we intraperitoneally injected groups of hamsters directly with 10% 263K BH. Terminal disease developed in all animals; the median incubation period was 151.4 (SE +30) days (Appendix Figure 2). We confirmed prion disease by biochemical detection of protease-resistant PrP (Figure 1, panel C). We did not detect a PrP^Sc^ signal in hamsters that did not show clinical signs, suggesting the absence of preclinical prion disease in those animals. Comparing incubation time and attack rate data obtained with WH and different dilutions of infected brain material suggests that the number of prions in each worm is equivalent to 1 × 10^−5^ to 1 × 10^−6^ dilution of infected brain. This estimation also is supported by analysis of the data by using a semiquantitative PMCA technique (*8*).

**Figure 2:**
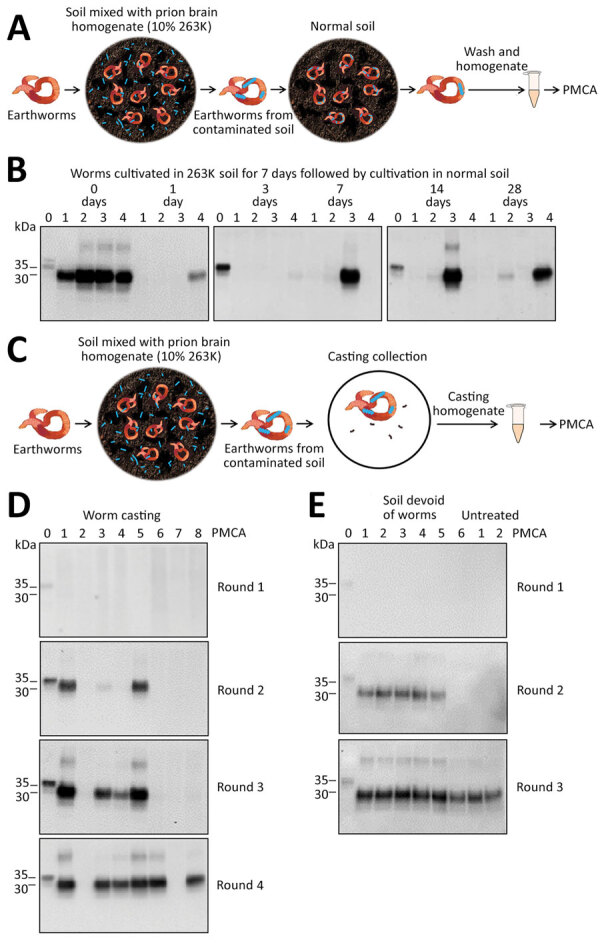
Detection of pathological prion protein (PrP^Sc^) retention and dispersion by earthworms. A) Process for exposing earthworms to PrP^Sc^–contaminated soil and analyzing for PrP^Sc^ retention. Worms were kept in PrP^Sc^–contaminated soil for 7 days, then transferred to normal, prion-free soil and collected at various times. After collection, worms were thoroughly washed, homogenized, and used for PrP^Sc^ detection. B) Western blot analysis of PMCA of worm samples after cultivation in 263K-contaminated soil for 7 days and exposure to normal soil for 0, 1, 3, 7, 14, and 28 days. Lane 0 is normal brain homogenate (NBH) used as positive control; lanes 1–4 indicate 4 different worms for each time point. C) Process for collecting castings excreted by prion-contaminated worms to analyze for PrP^Sc^. D) PMCA results for castings collected from earthworms exposed to 263K-soil for 7 days. Samples 1–8 were harvested and subjected to 4 PMCA rounds. E) Detection of PrP^Sc^ attached to 6 earthworms after exposure to prion-contaminated soil for 7 days. After collection and thorough washing, worms were dissected, and soil was carefully removed from the inside of the animal (soil-devoid worms). Worm carcasses were homogenized and used for PMCA detection of PrPS^c^. As controls, we used 2 untreated worms, that is, worms for which no soil was removed. In panels B, D, and E, all samples were digested with proteinase K (Sigma Aldrich, https://www.sigmaaldrich.com) at 50 µg/mL for 1 h at 37°C, except the NBH used as a migration control of PrP^C^. Numbers on the left indicate molecular weight markers. PMCA, protein misfolding cyclic amplification.

To investigate whether earthworms can retain infectious prions when exposed for different lengths of time to a prion-free environment, we exposed experimental subjects to prion-containing soil and subsequently transferred worms to naive soil (Figure 2, panel A). We collected worms from prion-containing soil after 7 days of exposure, thoroughly cleaned soil attached to the worms’ surface, and cultivated worms in naive soil for another 1, 3, 7, 14, and 28 days; we collected and analyzed 4 worms at each time point. PMCA results showed PrP^Sc^–positive signal for all 4 worms immediately after exposure to prion-contaminated soil (Figure 2, panel B). We found that 25%–50% of worms exposed to prion-free naive soil retained PMCA-detectable PrP^Sc^ (Figure 2, panel B). We observed no clear trend with the time of incubation in naive soil, and even animals exposed to prion-free soil for 28 days retained prions in their bodies (Figure 2, panel B).

To evaluate whether prion-contaminated earthworms excrete PrP^Sc^ back into the environment, we analyzed worm castings by using PMCA. We collected 2 worms exposed to prion-contaminated soil for 7 days and thoroughly washed worms with water. For casting collection, we placed animals in petri dishes and collected 8 pieces of casting from the petri dish to analyze PrP^Sc^ content by PMCA (Figure 2, panel C). The results showed 6/8 casting samples were positive for PrP^Sc^ (Figure 2, panel D). Of note, 3 samples had large amounts of PrP^Sc^ detectable by just 2 rounds of PMCA, indicating that earthworms exposed to prions in soil can take up and release PrP^Sc^ competent for prion replication.

Finally, to study whether some PrP^Sc^ molecules taken up from the soil remain attached to the body of the animal, we contaminated 6 worms by exposure to contaminated soil for 7 days. After washing to remove outside soil, we dissected animals to completely remove all soil particles inside the animal. We thoroughly washed worm bodies, homogenized them, and then used the homogenate for PrP^Sc^ detection by PMCA. Of the 6 six soil-void worms, 5 were positive for PrP^Sc^ after only 2 rounds of PMCA (Figure 2, panel E). The sixth worm became positive in the third PMCA round, as did control worms from which we did not remove internal soil (Figure 2, panel E). These results suggest that a substantial part of PrP^Sc^ taken up by worms from soil remained attached to the body of the animal and not merely in the soil particles that the worm acquired.

## Conclusions

The mechanisms implicated in the natural spread of infectious prions are not completely known. Some prion diseases, such as chronic wasting disease and scrapie, are thought to be highly transmissible through exposure to prion-contaminated environments (*2*,*3*). We previously demonstrated that infectious prions can attach to various components of the environment, including soil, plants, wood, and rock, and to several man-made surfaces, such as metals, plastic, and glass (*9*,*10*). However, little is known about how organisms living in the prion-exposed environment contribute to the spread of prions. In this study, we focused on earthworms (*E. fetida*) that live in close contact with known sources of prion infectivity in the environment, soil and diseased carcasses, and can move at a rate of 20–70 m/h (*11*,*12*). Our results demonstrate that earthworms can efficiently take up prions and act as vectors of prion disease transmission. In worms exposed to prion-contaminated soil, we noted PrP^Sc^ competent for both in vitro prion replication and in vivo infectivity. Even a relatively short exposure of 1 day was enough to contaminate all exposed worms. Of note, within 1 day after moving contaminated worms into prion-free soil, many earthworms were free of infectious particles. However, 25%–50% of worms retained PMCA-detectable PrP^Sc^ even 28 days after living in noncontaminated soil. Dissection of the worm’s bodies to separate tissue from soil inside the animal showed that a substantial amount of PrP^Sc^ was in the worm bodies. Furthermore, analysis of the casting excreted by contaminated worms showed that 75% of the animal feces contained a relatively large quantity of PrP^Sc^ detectable by PMCA. These results suggest that earthworms exposed to prions remain potentially infectious for long periods and release prions back into the soil, therefore possibly contributing to the spread of infectious prions in nature.

Appendix Additional information on uptake, retention, and excretion of infectious prions by experimentally exposed earthworms.
