# Supplementary material for: Uptake, Retention, and Excretion of Infectious Prions by Experimentally Exposed Earthworms
Source: Emerg Infect Dis. 2021 Dec;27(12):3151–4. doi: 10.3201/eid2712.204236 (PMC8632197; doi:10.3201/eid2712.204236)
Supplement: Appendix — Additional information on uptake, retention, and excretion of infectious prions by experimentally exposed earthworms. [file 20-4236-Techapp-s1.pdf]

Article DOI: https://doi.org/10.3201/eid2712.204236

Uptake, Retention, and Excretion of Infectious Prions by Experimentally Exposed Earthworms

Appendix

Materials and Methods

Earthworm Exposure

We acquired Earthworms (*Eisenia fetida)* from Orcon Organic Control Incorporation (https://organiccontrol.com) and exposed worms to contaminated soil as described below. We obtained and homogenized brain tissue from 263K Syrian golden hamsters (*Mesocricetus auratus*) showing advanced stages of prion disease, as previously described (*1*). We homogenously mixed 375 g of Elliot soil (a gift from Joel Pedersen, John Hopkins University) with 25 mL of 10% wt/vol 263K hamster brain homogenate (BH) prepared in phosphate-buffered saline (PBS) plus complete protease inhibitor cocktail (Roche, https://www.roche.com).

We placed earthworms on contaminated soil and collected at the indicated times. After collection, we thoroughly washed earthworms with tap water to remove soil attached to the surface of the worm and prepared a 10% wt/vol whole worm homogenate (WH) in PBS using a Precellys 24 tissue homogenizer (Bertin Technologies, https://bertin-technologies.com). We stored homogenates at –80°C until used for in vitro or infectivity assays. In addition, we obtained worm casting from animals exposed to prion containing soil for 7 days. For casting collection, we placed worms in disposable petri dishes for 2 h and then collected released castings.

Serial Prion Replication

We prepared substrate for protein misfolding cyclic amplification (PMCA) reactions as previously reported (*2*). In brief, we intracardially perfused 3–4-week-old female Syrian golden hamsters with PBS supplemented with 5 mmol EDTA. We made a preparation of 10% wt/vol

normal brain homogenate (NBH) in conversion buffer (150 mmol NaCL and 1% Triton X-100 in PBS).

To detect prion protein (PrP^{Sc}), we added 13 μL of WH to 117 μL NBH and loaded the mixture into 0.2 mL PCR tubes (USA Scientific, https://www.usascientific.com), each of which contained three 0.24-cm diameter Teflon beads (Hoover Precision Products, http://www.hooverprecisionplastics.com). We placed the PMCA tubes in a Q700 microsonicator (Qsonica, https://www.sonicator.com) and submitted tubes to PMCA cycles of 29 m 40 sec incubation at 37°C and brief 20 sec sonication at ≈260 watts. After a round of 96 cycles, we transferred 10 μL of the amplified samples into 90 μL NBH and performed another PMCA round until the detection limit was reached. To control the PMCA reaction, we serially diluted 10% wt/vol BH from 263K prion-infected animals into the PMCA substrate. Negative controls included samples containing PMCA substrate alone.

Inhibitory Effect of Worm Homogenate on PMCA Assay

We spiked 10% wt/vol whole and soil-cleansed prion-free WH to serial dilutions of 263K BH to a final concentration of 10^{-4} to 10^{-9} and then used this to seed the PMCA. We diluted the spiked WH 10 times into NBH and PMCA reactions started as described above. We compared the amplification efficiency to a control PMCA of 263K spiked NBH.

Proteinase K Digestion Assay and Western Blot Test

We were able to detect PrP^{Sc} by incubating aliquots of the sample with 50 μg/mL proteinase K (PK; Sigma-Aldrich, https://www.sigmaaldrich.com) for 1 h at 37°C and shaking at 600 rpm in a ThermoMixer (Eppendorf, https://www.eppendorf.com). After incubation, we added SDS-sample buffer (Invitrogen, ThermoFisher Scientific, https://www.thermofisher.com) and 33 mmol Dithiothreitol (DTT; Sigma-Aldrich) and heated samples at 95°C for 10 min. PK-resistant prion protein was fractionated by sodium dodecyl sulphate–polyacrylamide gel electrophoresis (ThermoFisher Scientific), transferred to a Hybond-ECL nitrocellulose membrane (Amersham GE Healthcare, https://www.gelifesciences.com) and detected by using 6D11 antibody (BioLegend, https://www.biolegend.com) diluted 1:5,000 in PBS/Tween. We visualized immunepositive bands by Amersham ECL Prime Western blotting detection kit (GE Healthcare) enhanced chemiluminescence assay (ECL) by using a ChemiDoc (Bio-Rad, http://www.bio-rad.com) image analysis system .

Infectivity Assay

Groups of 8–9-week-old female Syrian golden hamsters (Harlan Envigo, https://www.envigo.com) were intraperitoneally injected with 600 µL of 10% wt/vol homogenate of worms cultivated for 28 days in 263K contaminated soil. Homogenates from 3 different worms were used in this study. For negative controls, we used hamsters challenged with worms cultivated in NBH-treated soil. For positive controls, we directly injected hamsters interperitoneally with the 10% 263K BH. We pretreated positive and negative controls with ultraviolet radiation for 1 h to eliminate eventual bacterial contamination.

We monitored hamsters daily for signs of clinical disease. We measured progression of clinical signs by scoring the animals using a previously described system (*3*). We euthanized animals at advanced stages of prion disease, extracted brains and stored at –80°C. We humanely euthanized hamsters that did not develop clinical signs at 550 days after treatment and collected brains. We further confirmed prion disease by using biochemical analyses. All animal experimentation was performed following National Institutes of Health guidelines (*4*) and approved by the Animal Welfare Committee of the University of Texas Health Science Center at Houston.

References

1. Morales R, Duran-Aniotz C, Diaz-Espinoza R, Camacho MV, Soto C. Protein misfolding cyclic amplification of infectious prions. Nat Protoc. 2012;7:1397–409. PubMed https://doi.org/10.1038/nprot.2012.067

2. Pritzkow S, Morales R, Moda F, Khan U, Telling GC, Hoover E, et al. Grass plants bind, retain, uptake, and transport infectious prions. Cell Rep. 2015;11:1168–75. PubMed https://doi.org/10.1016/j.celrep.2015.04.036

3. Castilla J, Saá P, Hetz C, Soto C. In vitro generation of infectious scrapie prions. Cell. 2005;121:195–206. PubMed https://doi.org/10.1016/j.cell.2005.02.011

4. National Institutes of Health. Guide for the care and use of laboratory animals, eighth edition. Washington, DC: The National Academies Press; 2011.

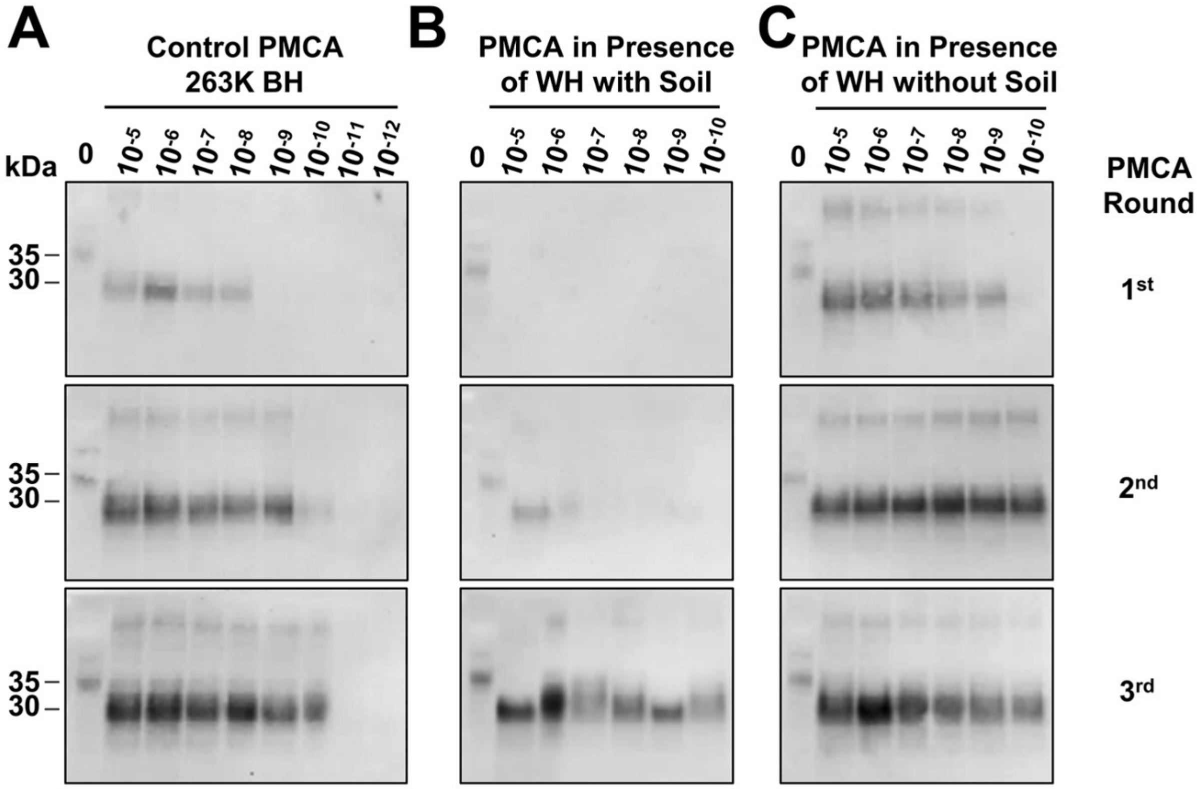

Appendix Figure 1. Results of dilution experiment using 263K hamster brain homogenate (BH) with and without worm homogenate (WH) to determine putative interference of worm and soil inside the animal body on the PMCA reaction. Samples were subjected to 3 serial rounds of 96 PMCA cycles and PrP^{Sc} signal was detected by Western blot after proteinase K (PK) digestion. For these studies, we used the same 263K BH as inoculum and wild type hamster BH as substrate. All samples were digested in PK except the normal brain homogenate (NBH) used as a migration control (lane 0). A) Control PMCA for amplification of PrP^{Sc} in 263K BH in the absence of WH. B) PMCA in the presence of 10% total WH without removing soil from inside worms before creating homogenate. C) PMCA in soil-devoid WH. No signal was ever detected in the absence of 263K BH (data not shown). For each reaction, lane 0 indicates NBH control and other lanes indicate homogenate dilution. Numbers on the left indicate molecular weight markers. Numbers on the right indicate PMCA round. PMCA, protein misfolding cyclic amplification.

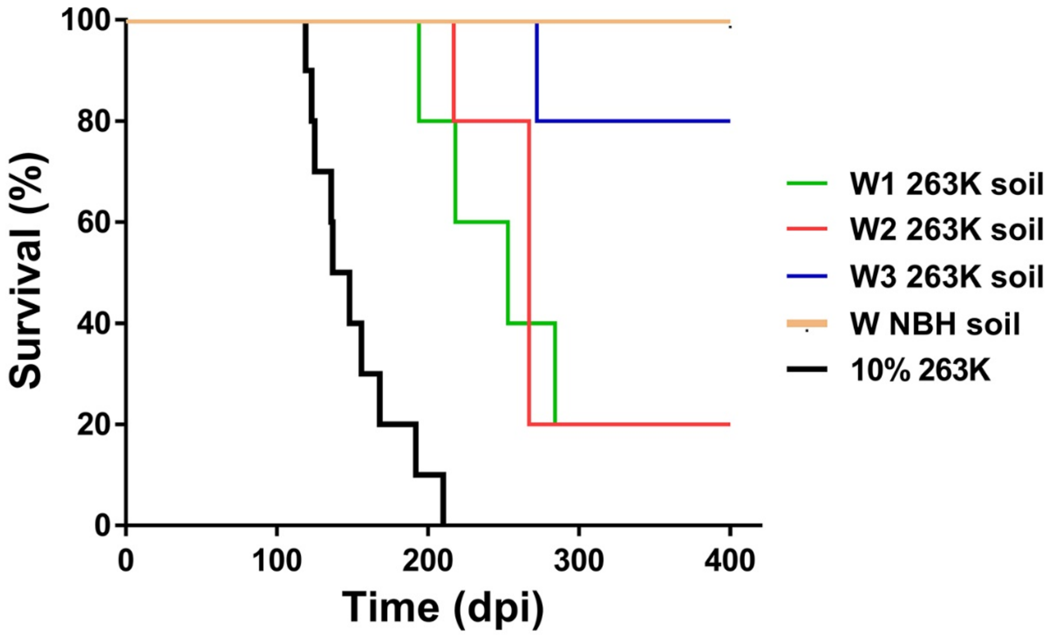

Appendix Figure 2. Survival curves for hamsters injected with homogenate from worms exposed to prion contaminated soil. Groups of 5 naive hamsters received intraperitoneal injection of 600 µL of 10% wt/vol WH derived from 3 different worms (W1, W2, W3). For a negative control (yellow line), 5 hamsters received intraperitoneal injection of 600 µL of a 10% wt/vol homogenate from a worm exposed to soil mixed with PrP^{Sc}–free brain extract. For a positive control (black line), 10 hamsters received intraperitoneal injection of 100 µL of 10% wt/vol brain homogenate from a 263K sick animal. Diseased animals exhibited typical 263K clinical signs, including ataxia, hyperactivity, aggression, and sensitivity to noise. Hamsters not showing signs of prion disease were humanely euthanized at 550 dpi. dpi, days post infection; NBH, normal brain homogenate; PrP^{Sc}, prion protein; WH, worm homogenate.
